# Pharmacists expand access to reproductive heaLthcare: PEARL study protocol

**DOI:** 10.1186/s12913-019-4038-9

**Published:** 2019-04-01

**Authors:** Maria I. Rodriguez, Blair G. Darney, Alison B. Edelman, Kimberly Yee, Lorinda B. Anderson, K. John McConnell

**Affiliations:** 1 Department of Obstetrics and Gynecology, Oregon Health & Science University, 3181 SW Sam Jackson Park Rd, UHN 50, Portland, OR 97239 USA; 2 Center for Health Systems Effectiveness, Oregon Health &Science University, Portland, USA; 3 College of Pharmacy, Oregon State University, Corvallis, USA

**Keywords:** Pharmacists, Hormonal contraception, Access to care, Continuation

## Abstract

**Background:**

In 2016, Oregon became the first of eight states to allow pharmacists to directly prescribe hormonal contraception (HC), including the pill, patch, or ring, without a clinic visit. In the two years following this policy change, the majority of ZIP codes across the state of Oregon had a pharmacist certified to prescribe HC.

**Methods:**

We will utilize complementary methodologies to evaluate the effect of this policy change on convenient access to contraception (cost, supply dispensed), safety, contraceptive continuation and unintended pregnancy rates. We will conduct a prospective clinical cohort study to directly measure the impact of provider type on contraceptive continuation and to understand who is accessing hormonal contraception directly from pharmacists. We will concurrently conduct a retrospective analysis using medical claims data to evaluate the state-level effect of the policy. We will examine contraceptive continuation rates, incident pregnancy, and safety measures. The combination of these methodologies allows us to examine key woman-level factors, such as pregnancy intention and usual place of care, while also estimating the impact of the pharmacist prescription policy at the state level.

**Discussion:**

Pharmacist prescription of HC is emerging nationally as a strategy to reduce unintended pregnancy. This study will provide data on the effect of this practice on convenient access to care, contraceptive safety and continuation rates.

## Background

Reduction of unintended pregnancy is important to both improve health and reduce public costs. Pharmacist prescription of hormonal contraception (HC) is a strategy that can potentially reach new contraceptive users and improve continuation rates among existing users by removing the barrier of a clinic visit. Pharmacist prescription of HC expands the scope of practice of pharmacists to screen women and to prescribe HC.

A national survey of women at risk of unintended pregnancy found that a majority of women (68%) liked the idea of accessing contraception directly from pharmacists, without a clinic visit first [1]. Other studies have established that women can self-screen and non-physicians can safely assess for medical contraindications to HC use [2–4]. One study suggested that contraceptive continuation may actually be improved with pharmacist prescription of contraception [5]. It is widely believed that because pharmacies do not require an appointment and have expanded hours and locations, direct HC access through pharmacies may improve contraceptive continuation rates over standard of care, which requires a clinic visit with a physician or advanced practice clinician for screening and prescription of the method, then a pharmacy visit to fill the prescription. Receiving HC directly from a pharmacist without a clinic visit is an innovative way to improve access to and continuation of contraception for women. This approach may be particularly relevant for disadvantaged populations of women who experience high rates of unintended pregnancy.

Beginning January 1, 2016, Oregon expanded the scope of practice of pharmacists to allow them to directly prescribe HC. This is a voluntary program, and pharmacists must complete five hours of education in order to be eligible to participate. The training program was available for pharmacists starting November 1, 2015. Pharmacists in Oregon demonstrated a high interest in prescribing contraception, and felt comfortable providing all covered methods after receiving training [6]. As of December 2018, 1340 pharmacists have been trained, and the majority of ZIP codes (64%) in the state of Oregon currently have a pharmacist certified to prescribe HC. Pharmacists can prescribe the pill, patch, ring or injection in Oregon. Pharmacists use a standardized screening tool and prescribing algorithm developed by the Board of Pharmacy [7].

Lessons learned from Oregon have national relevance: since Oregon’s program began, eight other states have introduced similar practices, all in the absence of evidence to guide implementation. We will examine who is using pharmacist prescription of HC and why. We will evaluate how the program impacts women’s experiences and satisfaction with contraceptive care. Our project will provide robust data on the role that pharmacist prescription of HC has on women’s convenient access to contraception (cost, supply dispensed), safety, contraceptive continuation and unintended pregnancy rates.

## Methods

### Study aims

To assess the impact of this policy change, we will use retrospective claims data combined with prospective primary data collection. We will compare all outcomes by prescriber type (pharmacist vs clinician). The study has the following specific aims.

Aim 1: Estimate the effect of pharmacist prescription of hormonal contraception (HC) on women’s convenient access to care.

Hypothesis 1a. Compared with clinician-only prescription, pharmacist prescription of HC will: 1) increase the number of women who are new users of HC, and 2) increase the supply of HC dispensed at each visit among reproductive age women with public and private insurance.

Hypothesis 1b. The average out-of-pocket payments for HC will not be different among women who receive HC from pharmacists compared with clinicians.

Aim 2: Compare the safety of HC prescriptions from pharmacists to the safety of prescriptions from clinicians.

Hypothesis 2a. Women with contraindications to estrogen use who receive HC from pharmacists will be less likely to be prescribed a combined (estrogen-containing) contraceptive compared to women who receive contraception from a clinician.

Hypothesis 2b. There will be no difference in rates of clinical follow-up between women who access HC in pharmacies compared with those who access HC from a clinician.

Aim 3: Determine the comparative effectiveness of pharmacist-prescribed HC on contraceptive continuation and pregnancy rate*s* at one year.

Hypothesis 3a. Women who receive HC from a pharmacist will be less likely to discontinue treatment in the next 12 months compared to women who receive HC from a clinician.

Hypothesis 3b. Women who receive contraception from a pharmacist will have fewer incident pregnancies (pregnancies per year) as compared with women prescribed HC by a clinician.

### Data sources

This project combines two approaches: a retrospective cohort study using medical claims data, and a prospective cohort study conducted at pharmacies.

#### Claims data

We will use the Oregon Health Authority’s All-Payer-All-Claims database (APAC). APAC includes administrative health care data for Oregon’s privately and publicly insured populations. APAC includes medical and pharmacy claims, non-claims payment summaries, member enrollment data, billed premium information, and provider identification information for all Oregonians who receive coverage through commercial insurers as well as through public payers (e.g Medicaid and Medicare). The APAC data provides a powerful tool to see the majority of insured individuals in the state. At any point in time, the database contains data for approximately 3.4 to 3.9 million individuals –representing about 87 to 98% of Oregon’s population (https://www.oregon.gov/oha/HPA/ANALYTICS/APAC%20Page%20Docs/APAC-Overview.pdf).

#### Prospective clinical cohort

We will enroll and follow a cohort of 500 women over one year. Clinical cohort recruitment will begin February 1, 2019. We will recruit women presenting to 160 retail pharmacies across Oregon, California, Colorado, Hawaii and Maryland for contraception, either prescribed by a clinician or a pharmacist. We will collect data at baseline and then at three, six and 12 months (four observations total over one year).

### Eligibility criteria

#### Retrospective cohort (claims data)

We will include data from January 1, 2014-December 31, 2018 to allow for 24 months of data before and 36 months of data after the policy change, a minimum of 600,000 medical and pharmacy claims. We will restrict the study population to reproductive-age girls and women (ages 12–51). Women who have had a hysterectomy, who are currently pregnant, or who are already using a long acting form of contraception (IUD, implant, tubal ligation) will be excluded from the analysis, since they are not candidates for HC use (not at risk of pregnancy). Exclusion criteria will be identified using relevant International Classification of Disease (ICD) 9 and 10 diagnosis and procedure codes.

#### Prospective clinical cohort

Women presenting for HC in a participating pharmacy, ages 18–51, who speak and read English and consent to participate are eligible. We will recruit 550 women to allow for a 10% loss to follow-up rate. We will collect three different points of contact (e.g. email, cell phone, work phone) for each participant to minimize loss to follow-up.

### Measures

This project has one key independent variable: prescriber type (clinician or physician). In the retrospective cohort (claims data), we will identify the prescriber associated with HC claims using National Provider Identifier (NPI) numbers. NPI is a unique identification number for covered health care providers and must be used in administrative and financial transactions under federal privacy law. While the NPI itself does not carry information about provider specialty, we will crosswalk it with a database of pharmacists in Oregon to classify providers as pharmacist or clinician. In the prospective clinical cohort, provider type will be self-reported on the baseline survey.

Outcome measures include: number of new HC users, number of months dispensed, woman’s out of pocket HC cost, safety (as measured by the number of women with estrogen contraindications who are using a progestin only method), accessing follow-up care, contraceptive continuation, and incident pregnancies. Table 1 lists outcome measures for each aim and data sources. We will contact participants via their preferred method to gather follow-up data on out-of-pocket costs, contraindications for estrogen, clinic visits related to reproductive health since the last interview, contraceptive continuation, pregnancy, pregnancy intentions during the next year, method switching, and prescriber-type switching.

**Table 1:** Summary of research aims and analysis plan sources

	Hypothesis	Measure	Source(s)	Outcome Definition
Aim 1 Convenient access to care	Aim 1a. Ease of access	New users of HC	Clinical cohort APAC cohort	Initiation of HC will be measured as a prescription for HC with no history of prior HC utilization in the preceding 30 days.
	Aim 1a. Ease of access	Number of months of contraceptive dispensed	Clinical cohort APAC cohort	Number of months and type of contraceptive coverage a woman receives per health system interaction.
	Aim 1b. Out of pocket payments	Amount a woman needs to pay per month of coverage	Clinical cohort	Amount a woman reports paying for care.
Aim 2 Safety	Aim 2a. Appropriate and safe prescription of HC for all women	Women with common contraindications to estrogen who are using a progestin only method	Clinical cohort APAC cohort	Contraindications will be identified as having received an ICD-9 or ICD-10 diagnosis code for hypertension, diabetes, migraines with aura, epilepsy, or blood clots in the last year on a claim in the last year. Pharmacy claims for these conditions will be identified with NDC codes.
	Aim 2b. Access to follow-up	Ability to access follow-up care	Clinical cohort	Utilization and location of follow-up after HC initiation.
Aim 3 Comparative Effectiveness	Aim 3a. Contraceptive continuation	Ongoing use of a contraceptive method	Clinical cohort APAC cohort	Ongoing use of contraceptive method (self-reported). No breaks in contraceptive claims of greater than 30 days. Missed days of contraception (self-reported). Gap of 3–29 days between the conclusion of one HC prescription and the fill of the next.
	Aim 3b. Pregnancy incidence	Pregnancies experienced by contracepting women	Clinical cohort APAC cohort	Self-reported pregnancy. Medical claims for any episode of pregnancy related care in study cohort using ICD9 &10 codes.

We will collect additional measures from each data source. For the retrospective cohort study, we will include the limited socio-demographic information available from claims data (age, race, ZIP code, and insurance type). In our prospective clinical cohort study we will collect an array of additional measures. Baseline data will include age, education, household income, insurance type, ZIP code, medical history, pregnancy history, previous contraceptive methods used, and motivation for seeking contraception directly from a pharmacist or with a clinician. We will specifically ask women about the amount of contraceptives received (e.g. pill packs or months of coverage). Participants will be asked at baseline if they intend to continue using their HC methods for 1 year, and asked at each follow-up encounter whether they have switched methods or provider type. Women will also be asked whether they are planning a pregnancy in the next year.

### Analytic procedures

#### General analysis plan

Each specific aim has a unique outcome (Table 1 and detailed below) and leverages both the prospective clinical cohort and the retrospective cohort. The key independent variable for all aims is prescriber type (pharmacist or clinician).

#### Plan for retrospective cohort (claims data)

All multivariable analyses will account for data clustering (non-independence of observations) at the clinical site level (pharmacy), individual level, and prescriber level (in the APAC, using a unique provider identifier) using robust standard errors, multi-level models, or fixed-effects approaches where appropriate. In addition, because women self-select into clinician or pharmacist provision of HC, we will assess differences in socio-demographic variables between treatment and comparison groups. If groups are not balanced, we will employ matching techniques, such as coarsened exact matching, which does not require specifying a model for exposure assignment, or propensity score weighting methods to adjust for imbalances between HC provision groups [2, 8–10]. This approach reduces bias due to self-selection into treatment and improves our inference about the relationship between pharmacist prescribing of HC and our outcome. We will pay special attention to the region of common support so our results will be interpretable for the entire treated group.

Our covariates will include age, race/ethnicity, rural/urban location, insurance, months of contraceptive dispensed, and other demographic factors that could also explain outcomes. Final models will be selected based on robustness to model specification and using standard methods of model selection such as the Bayesian Information Criteria (BIC) [11]. For all multivariable analyses described below, we will also calculate margins or predicted probabilities, which are easier to interpret than odds ratios or other measures of relative difference [12, 13]. Predicted probabilities will also aid in the assessment of the clinical and public health significance of our findings.

#### Plan for prospective clinical cohort

Our primary outcome for the clinical cohort study is contraceptive continuation rate at 12 months. Our sample size of at least 550 women will give us 80% power to detect a 12% difference in HC continuation rates at 12 months. Other secondary study outcomes are number of months dispensed, out of pocket cost to woman of HC, safety (measured by the number of women with estrogen contraindications who are using a progestin only method), accessing follow-up care, contraceptive continuation, and incident pregnancies.

In the clinical cohort, we are interested not only in the overall *population level effect* of pharmacist provision of HC, regardless of individual behaviors (intent to treat), but also in the *effect on those who did or did not switch* provider types (policy effect on the treated). We will identify women who report switching provider types - from clinician to pharmacist or vice-versa – at each follow-up interval. We will examine sample characteristics by baseline intent to continue and by reported switching to assess whether switching is associated with socio-demographic characteristics. Our longitudinal design means that provider type group assignment can be time varying – we will assign observations to the pharmacist or clinician group based on reported behaviors at the follow up data collection points. We will control for intent to continue HCs for 12 months at baseline as well as reported switching behaviors as potential confounders in multivariable analyses. For the population-level (intent-to-treat) assessment, we will classify women by group at baseline (clinician or pharmacist) and ignore subsequent switching behavior.

We will examine missingness in the prospective clinical cohort by comparing characteristics of those missing data and those not missing data to identify potential patterns in missingness (e.g. data not missing at random). If there is substantial missing data (more than 5% in a variable) in the clinical cohort study, we will conduct sensitivity analyses using multiple imputation approaches and compare those results with complete case results.

### Heterogeneity of treatment effects

Pharmacist provision of HC may impact some groups of women differently, in which case our average results will conceal important information. We want to understand how this intervention affects contraceptive services across all women, and for particular vulnerable subpopulations (e.g. adolescents). Therefore, heterogeneity of treatment effects is central to our study. For all analyses we will conduct additional exploratory analyses: stratification and/or interaction by key demographic variables information available in the retrospective APAC cohort and in the prospective clinical cohort (age, race/ethnicity, rurality, insurance status) to assess if the effect of the policy intervention was modified by group membership. We will pay special attention to subgroups with known barriers to access and disparities in health outcomes [14–17].

## Discussion

Unintended pregnancy has remained an entrenched public health problem in the United States with multigenerational health consequences and significant public costs. Pharmacist prescription of HC is a health system innovation that has the potential to reduce unintended pregnancy, and is a practice that is rapidly emerging nationally. Oregon was the first state to implement this policy change in January 2016, and since then, seven additional states have implemented the program. Scant data exists to understand how pharmacist prescription of HC impacts women’s experience of care, contraceptive safety and efficacy. Our study will decrease this gap in the evidence by documenting the implementation of Oregon’s policy and evaluating how it has impacted reproductive health outcomes.

This study is not without limitations. It reflects the experience of one state with pharmacist prescription of HC, and outcomes may vary for settings where the program was implemented differently. For example, Oregon’s Medicaid has reimbursed for pharmacist time in counseling and the cost of the drug since the program start. Other states have not required insurers to reimburse pharmacists for the time spent in counseling. Additionally, not all states permitting pharmacist prescription of HC require additional training, which may impact uptake of this voluntary practice among pharmacists. Our clinical cohort study will collect data on pregnancy intention among participants, but is not powered to examine differences in unintended pregnancy rates. We will be able to use the larger sample size in claims data to examine incident pregnancies among current contraceptive users as a proxy for unintended pregnancy at the state level.

Pharmacist prescription of contraception is an emerging practice nationally, and evidence is needed to determine the acceptability, safety and efficacy of this policy. Our findings on the impact of Oregon’s program will have relevance for states nationwide that are beginning similar programs.

## Abbreviations

APAC: All Payor All Claims
BIC: Bayesian Information Criteria
GEE: Generalized estimating eq.
HC: Hormonal contraception
ICD: International Classification of Disease
NPI: National Provider Identifier
